# Benznidazole as Prophylaxis for Chagas Disease Infection Reactivation in Heart Transplant Patients: A Case Series in Brazil

**DOI:** 10.3390/tropicalmed5030132

**Published:** 2020-08-18

**Authors:** Joao Manoel Rossi Neto, Marco Aurelio Finger, Carolina Casadei dos Santos

**Affiliations:** Instituto Dante Pazzanese de Cardiologia, Av Dante Pazzanese 500, Sao Paulo CEP 04012-909, Brazil; mfinger@uol.com.br (M.A.F.); htx@dantepazzanese.org.br (C.C.d.S.)

**Keywords:** chagasic cardiomyopathy, heart transplant, chagas disease reactivation

## Abstract

Background—Patients with Chagas cardiomyopathy (CC) have high mortality, and CC is a common indication for heart transplantation (HTx) in endemic countries. Chagas disease reactivation (CDR) is common after transplantation and is likely to cause adverse outcomes unless detected and treated appropriately. This study reviews our experiences with HTx among patients with CC, and the use of benznidazole (BZ) before transplantation. Methods—During the 18-year period from 1996 through 2014, 70 of 353 patients who underwent HTx (19.8%) had CC, and 53 patients met the inclusion criteria. The effectiveness of prophylactic treatment with BZ (dose of 5 mg/kg/day, two times per day, for at least four weeks and for a maximum of eight weeks) was determined based on the observed reduction in the incidence of CDR during the post-HTx period. Results—Prophylactic therapy was administered to 18/53 patients (34.0%). During the follow-up period, the incidence rate of CDR in our study was 34.0% (18/53). Based on logistic regression analysis, only prophylaxis (OR = 0.12; CI 0.02–0.76; *p* = 0.025) was considered to protect against CDR. Conclusion—Our study suggests that the use of BZ may reduce the incidence of CDR in patients undergoing HTx and warrants further investigation in a prospective, randomized trial.

## 1. Introduction

Chagas disease (CD) is a major cause of end-stage cardiomyopathy in Mexico, South America, especially in Brazil, and Central America, with 7.7 million people currently estimated to be infected in 18 countries [1]. The exodus from these rural communities in the late 20th century has resulted in a marked urbanization and globalization of CD. The number of infections in the United States and non-endemic countries in Europe and the Western Pacific Region continues to rise [2,3].

Patients with chagasic cardiomyopathy (CC) have higher mortality than patients with other etiologies of cardiomyopathy [4]; thus, CC is a common indication for heart transplantation (HTx) in endemic countries where this therapy is available.

Chagas disease reactivation (CDR) is common after HTx and is likely to cause adverse outcomes unless detected and treated appropriately [5]. In addition, acute *T. cruzi* infection causes substantial morbidity (allograft dysfunction) and mortality in the post-transplant setting if not recognized and treated early [6]. 

Only two drugs (benznidazole (BZ) and nifurtimox) have been shown to be effective enough to warrant widespread use in acute CD treatment [7]. Data validating the use of prophylactic therapy with BZ in patients with CD who underwent heart transplantation are lacking in the literature. 

In 1995, Fragata Filho [8] suggested the necessity of elimination of the parasite with parasiticidic drugs in patients with a heart transplant and immunosuppression therapy. In addition, in 1999, Rassi [9] described the protective effect of benznidazole (5 mg/kg/day, two times per day, 4–8 weeks) against parasite reactivation in patients chronically infected with *T. cruzi* and treated with corticoids for associated diseases. Based on these assumptions, our service began using BZ as prophylactic therapy.

This study reviews our experience with cardiac transplantation in patients with CC, emphasizing reactivation and the use of BZ before transplantation.

## 2. Materials and Methods 

### 2.1. Patients and Medications

This investigation is a retrospective cohort study of heart transplant recipients with CC that was undertaken at Dante Pazzanese Institute of Cardiology and includes data compiled over a 18-year period. Between 1996 through 2014, 70 patients with CC underwent HTx. Seventeen patients were excluded because they died during hospitalization or had incomplete data. Clinical data were considered until December 2016 to guarantee at least 2 years of follow-up after transplantation.

All patients were evaluated for CD using serological screening before starting conditioning chemotherapy and transplantation. 

All patients received triple drug immunosuppressive therapy that included calcineurin inhibitors (cyclosporine or tacrolimus), corticosteroids, and a third drug, which could be mycophenolate mofetil or sodium. Due to the lack of BZ that usually occurred during this period, BZ was used only when it was available and in a non-randomized way. Prophylactic therapy with BZ was prescribed at a dose of 5 mg/kg/day, two times per day, for at least 4 weeks and for a maximum of 8 weeks [9].

The protocol was approved by the review boards of the Instituto Dante Pazzanese de Cardiologia (CAAE: 47457315.8.0000.5462).

### 2.2. Definitions

The criteria used to diagnose CDR after HTx were evidence of parasites obtained using direct methods, such as endomyocardial biopsies (EMB), hemoculture or real-time polymerase chain reaction (PCR). None of the donors had CD.

The effectiveness of the treatment was determined based on the observed reduction in the incidence of CDR after heart transplantation.

### 2.3. Statistical Analysis

Continuous variables are expressed as the mean ± standard error (SE), and categorical variables are expressed as percentages. For group comparisons, the *t*-test was used for variables with a normal distribution and the Mann–Whitney test was used to compare variables without a normal distribution. For categorical variables, the χ^2^ test or the Fisher exact test was applied. For the multivariate analysis, the logistic regression model was used with the Hosmer and Lemeshow test. *p* < 0.05 was considered significant. All analyses were performed using GNU PSPP (Version 0.8.5-g2d71ac) (Computer Software). Free Software Foundation. Boston, MA, USA.

## 3. Results

From 1996 through 2014, 353 heart transplantations were performed at Dante Pazzanese Institute of Cardiology. Seventy (19.8%) transplants were performed in patients with CC. Table 1 shows the observed differences in characteristics between the groups without prophylactic therapy (GP-) and with prophylactic therapy (GP+).

Prophylactic therapy was administered in 18 patients (34.6%). Of the 18 (34.6%) patients with CDR, the diagnosis was made using EMB (44.4%), PCR (38.9%), and hemoculture (16.7%). The main symptoms in the clinical presentation included dyspnea (33.3%), weakness (27.7%), vomiting/diarrhea (16.6%), heart failure (11.1%), and no symptoms (11.1%).

The total incidence rate of CDR in our study was 34.0% (18/53). CDR was diagnosed in 45.7% (16/35) of patients without prophylaxis and in 11.1% (02/18) of patients in the group with prophylaxis (*p* = 0.012). The mean time for CDR was 352 days (SE = 148.4).

The univariate analysis of the characteristics of patients with (CDR+) and without (CDR-) Chagas disease reactivation (Table 2) shows that both cyclosporine (*p* = 0.041) and prophylaxis (*p* = 0.012) were significant, but in the multivariate analysis, which also included variables that we thought could influence the reactivation results, only prophylaxis remained significant (*p* = 0.025) (Table 3).

## 4. Discussion

CDR has been described in patients with leukemia and HIV and in patients with kidney, liver, and heart transplantation. It is important to highlight that due to immunosuppression, the recipients with CD can develop CDR, and the reactivation rate of CD after HTx for CC is variable, with an incidence of 21–45% [5]. In the literature, the degree of immunosuppression was often correlated with reactivation [6,7]. At our institution, reactivation occurred in 34.6% of patients with CC. The type and dose of immunosuppression used did not differ significantly between the groups with and without CDR. Unlike the Bacal [10] study that showed that mychophenolate mofetil increased CDR in heart transplanted patients, our data failed to demonstrate which immunosuppressant could increase the chance of CDR. We hypothesized that prophylaxis might have a protective effect or that our small sample size was not powered enough to determine the effects of the immunotherapy.

Nevertheless, CDR can cause both cardiac and noncardiac (skin lesions) manifestations. The cardiac manifestations of CDR include new conduction blocks that may require pacemaker placement, valvular regurgitation, and most seriously, allograft dysfunction that can progress to cardiogenic shock and death [5,7]. In the setting of CDR after HTx, we found great clinical variability, from patients with no symptoms to patients with heart failure.

In Brazil, the most recent Clinical Protocol and Therapeutic Guidelines for Chagas Disease of the Ministry of Health [11] stated that the treatment of the acute form must be immediate. In asymptomatic cases, or when diagnostic confirmation is not possible, but with persistent suspicion, empirical treatment may be considered. The etiological treatment of the person affected with the disease in the chronic phase should be carried out in children and adolescents in the chronic indeterminate phase. For adults in the chronic indeterminate phase, the benefit of antiparasitic treatment is uncertain. In adults under 50 years old, the treatment should be considered. For Chagas heart disease in the early stages (only have changes in the electrocardiogram, with normal ejection fraction, absence of heart failure, and absence of severe arrhythmias), both treatment and untreatment with benznidazole are valid alternatives, and the decision must be shared with the patients. There is no evidence to justify the treatment of patients with advanced Chagas heart disease [11].

Prophylaxis has been demonstrated to avert transmission or reactivation in liver and renal recipients [12,13]. In patients with CC who are treated with corticoids, Rassi [9] demonstrated that for many reasons, benznidazole used as a primary chemoprophylaxis prevented increased parasitemia (evaluated by xenodiagnoses) and suggested that in immunocompromised patients with chronic CD, the use of this drug could be useful. Importantly, the myocarditis of an acute CDR patient can be easily mistaken for allograft rejection [14], and treatment with intensified immunosuppression can lead to more severe *T. cruzi* infection [7]. Unfortunately, due to a lack of raw materials or difficulty obtaining the medication for prophylactic use, BZ was unavailable for some of the study period, making it impossible to carry out a randomized study in our center.

When we started our study, PCR was not considered a consolidated diagnosis of Chagas reactivation. Since then, new scientific data have demonstrated advantages of PCR monitoring over traditional methods such as being less invasive, faster, contributing to the differential diagnosis between reactivation and rejection episodes, and preceding the clinical manifestations of reactivation by 2 or more months [15,16,17,18]. Thus, PCR is a precious tool which guides physicians to decide whether patients should begin receiving anti-parasite drugs or changes in the immunosuppression regimen. Today, the concept of reactivation must be redefined even if the patient is asymptomatic and includes an increase in parasitemia, detected either by direct parasitological techniques or by PCR [7].

To our knowledge, this study is the first reported series using BZ as prophylactic therapy before HTx in patients with CC. This study found that in the presence of prophylactic therapy, reactivation was reduced in patients with CC who were subjected to HTx (OR = 0.12, CI 0.02–0.76; *p* = 0.025).

Our findings reinforce the idea that monitoring for CDR should be performed routinely after HTx and during suspected reactivation episodes [7,19]. However, no scientific data are available that define exactly when such routine monitoring should be performed.

Our study has limitations common to retrospective analyses. First, the individuals enrolled in the study were not randomized to BZ therapy or placebo, and the physicians and patients were not blinded to the medical intervention. In addition, this study represents an analysis of a unique center with a small number of patients.

## 5. Conclusions

Our results suggest that the use of prophylactic therapy could reduce the incidence of CDR in patients who were subjected to HTx. These findings challenge the available data and warrant further randomized, double-blind studies.

## Figures and Tables

**Table 1:** Characteristics of patients according to benznidazole prophylactic therapy status (GP+ vs. GP−).

Variable	Total *n* = 53	GP− *n* = 35	GP+ *n* = 18	*p*-Value
**Male (%)**	32 (59.6)	19 (54.3)	13 (72.2)	0.261
**Reactivation (%)**	18 (34.0)	16 (45.7)	02 (11.1)	0.012
**Corticosteroid therapy (%)**	39 (75.0)	28 (80.0)	11 (61.1)	0.195
**Tacrolimus therapy (%)**	04 (7.5)	03 (08.6)	01 (05.6)	0.616
**Cyclosporine Therapy (%)**	31 (58.5)	22 (62.9)	08 (44.4)	0.252
**Mycophenolate (%)**	37 (69.8)	26 (74.3)	11 (61.1)	0.401
**Age in years (m; SE)**	48.8 (1.38)	50.4 (1.55)	46.0 (2.72)	0.141
**Weight in Kg (m; SE)**	62.1 (1.53)	61.6 (1.87)	62.9 (2.56)	0.671
**Corticosteroid in mg (m; SE)**	22.1 (2.62)	20.1 (3.30)	24.7 (4.27)	0.396
**Tacrolimus in mg (m; SE)**	7.0 (1.30)	7.3 (1.76)	6.0 (-)	0.742
**Cyclosporine in mg (m; SE)**	220.2 (11.21)	208.8 (18.06)	233.9 (11.30)	0.272
**Mycophenolate in mg (m; SE)**	1055.5 (59.08)	1058.7 (61.36)	1050.7 (119.55)	0.948
**Ischemic time in min (m; SE)**	146.2 (7.65)	152.0 (10.32)	134.8 (7.37)	0.333

m = mean; SE = standard error.

**Table 2:** Characteristics of patients with (CDR+) and without (CDR−) Chagas disease reactivation.

Variable	CDR− *n* = 35	CDR+ *n* = 18	*p*-Value
**Male (%)**	21 (58.3)	11 (61.1)	0.938
**Prophylaxis (%)**	16 (45.7)	02 (11.1)	0.012
**Corticosteroid therapy (%)**	23 (65.7)	16 (88.9)	0.700
**Tacrolimus therapy (%)**	03 (08.6)	01 (05.6)	0.694
**Cyclosporine Therapy (%)**	17 (48.6)	14 (77.8)	0.041
**Mycophenolate (%)**	23 (65.7)	15 (83.3)	0.177
**Age in years (m; SE)**	49.3 (1.73)	47.8 (2.32)	0.626
**Weight in Kg (m; SE)**	61.7 (1.93)	62.9 (2.56)	0.703
**Corticosteroid in mg (m; SE)**	20.1 (3.30)	24.7 (4.27)	0.396
**Tacrolimus in mg (m; SE)**	7.3 (1.76)	6.0 (-)	0.742
**Cyclosporine in mg (m; SE)**	208.8 (18.06)	233.9 (11.30)	0.272
**Mycophenolate in mg (m; SE)**	1058.7 (61.36)	1050.7 (119.55)	0.948
**Ischemic time in min (m; SE)**	148.0 (10.20)	144.4 (10.94)	0.826

m = mean; SE = standard error.

**Table 3:** Logistic regression for CDR *.

	B	S.E.	Wald	df	Sig.	Exp(B)	Lower	Upper
**Gender**	−1.15	0.79	2.13	1	0.144	0.32	0.07	1.48
**Prophylaxis**	−2.13	0.95	5.04	1	0.025	0.12	0.02	0.76
**Corticosteroid therapy**	0.73	1.04	0.49	1	0.482	2.07	0.27	15.86
**Tacrolimus therapy**	−0.38	1.56	0.06	1	0.805	0.68	0.03	14.41
**Cyclosporine therapy**	1.16	0.93	1.56	1	0.212	3.19	0.52	19.69
**Mycophenolate therapy**	0.97	0.95	1.04	1	0.308	2.63	0.41	16.90
**Constant**	−0.57	1.24	0.21	1	0.648	0.57		

* Hosmer and Lemeshow test = 0.620.
